# Defect Detection of Adhesive Layer of Thermal Insulation Materials Based on Improved Particle Swarm Optimization of ECT

**DOI:** 10.3390/s17112440

**Published:** 2017-10-25

**Authors:** Yintang Wen, Yao Jia, Yuyan Zhang, Xiaoyuan Luo, Hongrui Wang

**Affiliations:** 1 School of Science and Technology, Yanshan University, Qinhuangdao 066004, China; ytwen@ysu.edu.cn; 2 School of Electrical Engineering, Yanshan University, Qinhuangdao 066004, China; jiayao89@sina.cn (Y.J.); yyzhang@ysu.edu.cn (Y.Z.); hb_wang@ysu.edu.cn (H.W.)

**Keywords:** thermal insulation material, electrical capacitance tomography, defect detection, image reconstruction, PSO

## Abstract

This paper studies the defect detection problem of adhesive layer of thermal insulation materials. A novel detection method based on an improved particle swarm optimization (PSO) algorithm of Electrical Capacitance Tomography (ECT) is presented. Firstly, a least squares support vector machine is applied for data processing of measured capacitance values. Then, the improved PSO algorithm is proposed and applied for image reconstruction. Finally, some experiments are provided to verify the effectiveness of the proposed method in defect detection for adhesive layer of thermal insulation materials. The performance comparisons demonstrate that the proposed method has higher precision by comparing with traditional ECT algorithms.

## 1. Introduction

Thermal insulation materials are widely used in aeronautics and astronautics for their characteristics, such as light weight and heat insulation, etc. [1]. At present, the thermal insulation materials are usually glued to the surface of spacecrafts by adhesive. In the complex space environment, the adhesive layer defects of thermal insulation materials, such as cracks and bubbles in the rubber may cause the thermal insulation materials broken off during flying, and hence, it is important to detect the adhesive layer defects of thermal insulation materials for spacecraft safety. Along with the development of new adhesive processes, traditional defect detection technologies no longer satisfy the high accuracy requirements [1,2,3]. Therefore, developing new techniques and methods to detect defects of adhesive layer for thermal insulation materials is an urgent work.

Recently, some interesting defect detection methods for thermal insulation materials and composite materials with perfect physical properties, e.g., material uniformity, electrical conductivity, etc. have been reported. For example, Park and Kyu [2], Sun and Zhou [3] presented a method based on laser ultrasonic technology for defect detection of carbon fiber resin matrix composites pore fastening holes and composite materials of high temperature resistant layers. Guo and Jing proposed a method based on infrared thermal wave NDT for debonding flaws in some helicopter blades [4], which was analyzed by the thermogram and the peak amplitude image of the second derivative thermogram. A method for coating thickness testing and internal defects detection based on infrared thermal wave was developed in [5], where the interior defects can be detected through measuring the thickness of samples with infrared thermal wave. A method to find counterfeit drugs quickly and reliably based on transmission spectroscopic terahertz (THz) measurement technique was developed in [6]. Palka and Krimi [7], Li and Ding [8] proposed a method based on terahertz time-domain spectroscopy for thickness detection of composite materials, which can obtain the thicknesses of all of the layers of the composite materials based on a time-domain fitting procedure. A method based on laser ultrasonic detection of drilling-induced delamination was presented for the test of composite laminates in [9], where the laser ultrasonic C scan was used to test composite laminates, and the morphologies, dimensions, and positions of drilling-induced delamination can be obtained. Zhang and Gao proposed a method that applied wavelet transform and fuzzy pattern recognition to ultrasonic detection [10]. In this way, they can detect the bonding quality for thin composite plate.

The effectiveness of the aforementioned detection methods have been verified in some application fields. However, some limitations are obvious in the field of defect detection for adhesive layer of thermal insulation materials. Firstly, ultrasonic test is less satisfactory in performance for the insulation materials that have loose structures or uneven characteristics. Secondly, infrared thermal wave test cannot penetrate the adhesive layer defect for the materials which have strong heat-resistant. In other words, infrared thermal wave was unable to effectively obtain the information of adhesive layer of thermal insulation materials. Thirdly, terahertz test is greatly influenced by the characteristics of thermal insulation materials. In addition, terahertz test is poor in obtaining the information of the layer of medium distribution.

It is necessary to develop a new method for defect detection of adhesive layer of thermal insulation materials. Towards this end, a defect detection method based on electrical capacitance tomography is proposed in the paper. A detection system of planar electrode capacitance is adopted, and an Improved Particle Swarm Optimization (IPSO) algorithm is proposed for the defect detection of adhesive layer of thermal insulation materials. Then, an experiment of defect detection of the bonding layer of thermal insulating materials is provided to verify the effectiveness of the proposed defect detection algorithm. The obtained results demonstrate that the proposed defect detection method has higher performance than the traditional Electrical Capacitance Tomography (ECT) methods.

## 2. Defect Detection Principle of ECT

**Figure 1:**
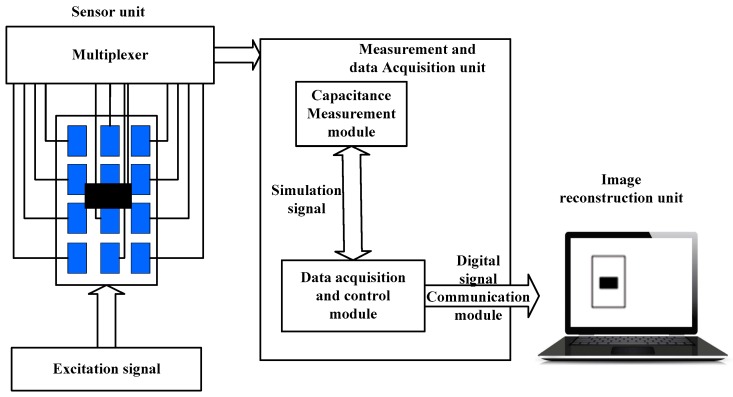
Electrical Capacitance Tomography (ECT) system structure diagram.

ECT is a new nondestructive testing technology developed in recent years based on the mechanism of capacitance sensitive, and it has been widely applied in the fields of industrial fluidized bed monitoring, multidirectional flow detection, and medical science [8,9]. The basic principle of ECT imaging technology is that the multiphase medium often has different dielectric constants, such that the medium distribution images can be obtained through capacitance sensors. In this paper, according to the dielectric properties of rubber insulation materials and the characteristics of material surface structure, planar capacitive electrode substrate is used. As shown in Figure 1, an ECT system contains three modules: capacitance sensor module, measurement and data acquisition module, and image reconstruction module. The working process of the ECT system is as follows: acquire the capacitance values via capacitance sensors firstly, and then transmit the values to the computer, and finally reconstruct the field distribution image in computer.

Planar electrode plates of 12 electrodes are used in the capacitance sensor unit. In order to guarantee the credibility and accuracy of measurement data, shielding processing is adopted between the electrodes, and the interface of detection electrode, as well as in the data acquisition unit. The different active electrodes are selected by a multiplexer. The capacitance measurement system of the Intertek Testing Services (ITS) company is used in the measurement and data acquisition unit for experimental data processing of capacitance plate collection. The sensitivity field of material distribution is generated by using the software of ANSYS.

## 3. Reconstruction Algorithm of Image

Reconstruction algorithm of image is to use the collected data from the measurement and data acquisition module of ECT system to build the image projection, and then one can obtain the field distribution diagram, which is used to defect detection and defect analysis for thermal insulation materials. Two key computational problems are required to be solved in ECT: the forward problem and the inverse problem. For the forward problem, inter-electrode capacitances are to be determined by the permittivity distribution. In this paper, a planar capacitive sensor array containing *n* = 12 electrodes is used, and then one has *M* = *n*(*n* − 1)/2 = 66 independent capacitance measurements.

Without loss of generality, the effect of shielding layer to dielectric capacitor is neglected, and then based on the electrical principle, the capacitance can be computed as follows [10]

(1) $$C_{i}=\iint_{D}\varepsilon (x,y)\cdot S_{i}(x,y,\varepsilon (x,y))dxdy,\;i=1,2,\cdots ,66$$

where *D*, ε(x,y) and $S_{i}(x,y,\varepsilon (x,y))$ are the electrode surface, the permittivity distribution of sensing field, and the sensitivity matrix of the sensor’s imaging field, respectively. The capacitance differences, which are produced by different material properties on tiny pixel areas, can be distinguished by sensitivity matrix. If we segment the material small enough, then the function of sensitivity distribution is affected slightly by medium distribution [10], and thus Equation (1) can be simplified as follows

(2) $$C_{i}=\iint_{D}\varepsilon (x,y)\cdot S_{i}(x,y)dxdy$$

where $S_{i}(x,y)$ is the sensitivity function of material capacitance $C_{i}$. Then, one can linearize and discretize Equation (2), as follows

(3) C=SG

where **C** denotes the normalized capacitance vector, **G** is the normalized permittivity vector, and **S** represents the normalized sensitivity matrix. Thus, the forward problem is modeled by Equation (3).

For the inverse problem, one needs to acquire the permittivity distribution based on capacitance measurements. Usually, the result of this problem is shown by a visual image, and thus this process is also called image reconstruction.

If there exists the inverse of matrix **S**, we can solve Equation (3) directly by

(4) $$S^{-1}C=G$$

Unfortunately, the matrix **S** cannot be obtained accurately, because there are three major difficulties for the inverse problem. The first one is the “soft field characteristics [11]”, i.e., the measurement sensitive field of ECT sensor is affected greatly by medium distribution. The second one is that Equation (4) is an indeterminate equation, since the number of unknown variables N (i.e., the number of pixels) is usually much larger than the number of equations M (i.e., the number of capacitance measurements), and thus the solution is not unique. The third one is that Equation (4) is an ill conditioned equation [12].

In the past few years, a number of image reconstruction algorithms have been developed to address the ill posed and ill conditioned problems. In general, they can be categorized into two groups: non-iterative (or single step) algorithms (e.g., Tikhonov Algorithm [9], Linear Back Projection (LBP) Algorithm [10], etc.), and iterative algorithms (e.g., SIRT Algorithm [12], Landweber Algorithm [13], etc.). Here, we introduce two typical algorithms.

### 3.1. LBP Algorithm

Linear Back Projection (LBP) is a non-iterative algorithm and it is the earliest algorithm for ECT imaging technology [10], where if **S** is considered to be a linear mapping from the permittivity vector space to the capacitance vector space, $S^{T}$ can be considered as a related mapping from the capacitance vector space to the permittivity vector space. Then the approximated solution can be given as follows.

(5) $$G=S^{T}C$$

LBP algorithm is still widely used for on-line image reconstruction because of its simplicity. However, it produces poor-quality image and can only provide qualitative information. LBP algorithm is commonly used in qualitative analysis. However, for complex media distribution error detection, its resolution accuracy for image reconstruction is relatively low.

### 3.2. Landweber Algorithm

The Landweber algorithm [13] is an iterative algorithm and is developed based on the foundation of steepest descent method. Up to now, the Landweber algorithm has been widely used in the field of ECT. The main principle is to correct the solutions of the equation in the minus gradient direction of data residuals. The data residual gradient is shown as follows

(6) $$\nabla \cdot \frac{1}{2}{\left\|SG-C\right\|}^{2}=S^{T}(SG-C)$$

and the iterative equation is

(7) $$G^{\left(k+1\right)}=G^{\left(k\right)}+\alpha S^{T}(C-SG^{\left(k\right)})$$

where α is the positive scalar, which plays an important role in the process of iteration. However, **G** ($G=S^{T}C$) is regarded as the initial guess in the process of iterative calculation, which will produce a large error between the initial guess and real value [12]. Traditional ECT image reconstruction (either the iteration or non-iteration) is flat of sensitivity field. However, in practical applications, phase distribution of different types may cause the differences of sensitivity field. If the differences are ignored, the accuracy of defect detection will be influenced seriously.

### 3.3. Defect Detect Algorithm Based on Improved PSO

#### 3.3.1. Data Processing Based on LS-SVM

In ECT system, the measuring capacitance C will have relatively subtle change for the small defects of adhesive layer of thermal insulation material; besides, the sensitivity matrix S is generally considered to be constant, which will result in certain errors in the rubber with different defects. Thus, the experimental value of the capacitance $C_{m}$ has a certain deviation in the calculative value of the formula. The deviation is given as follows

(8) $$\Delta C=C_{m}-S\cdot G$$

where $C_{m}$ is the normalized measurement value of the capacitance [13], and **G** is the matrix of dielectric constant distribution.

By using multiphase medium with different permittivities, the image of medium distribution can be obtained through measuring the obtained permittivities by capacitance sensors.

The errors between capacitance measurements and theoretical simulation capacitance values can be obtained through training samples, which are trained by least squares support vector machine (LS-SVM).

The vector norm of Equation (8) is as follows

(9) $$y=\left\|\Delta C\right\|=\left\|C_{m}-S\cdot G\right\|=f(x):R^{n}\to R^{1}$$

where $R^{n}$ is the *n* dimensional real vector set and $R^{1}$ is the real set.

In Equation (9), the measurement capacitance can be viewed as the input vector while the norm of the difference vector between the measurement capacitance vector and the computed capacitance vector is viewed as the output vector.

As training samples used by LS-SVM, the measurement capacitance vectors of defects are used as the input samples, and the norm of capacitance deviation vectors of the same defects are used as the output samples. According to the theory of SVM, the more the training samples are used, the stronger the generalization ability is [14]. In this paper, three kinds of common defects of composite material bonding structure (i.e., fracture defect, bubbles, lack of glue) are considered as a sample set, where each kind of defects have 32 samples with each sample has only one type defect at a particular position, and totally 96 training samples are used.

#### 3.3.2. Image Reconstruction Algorithm Based on Improved PSO

Particle swarm optimization (PSO) algorithm was first proposed in 1995 by the American social psychologist James Kennedy and electrical engineer Russell Eberhart [15]. After that, some other similar algorithms were further proposed. In these algorithms, the evolution of PSO algorithm is also used by the concept of “community” and “evolution”. It is also based on the fitness of individuals (particles) size in these algorithms. The difference is that the particle swarm algorithm to each operator as in *n* dimensional search space does not have a weight and volume of small profit, and in the search space at a certain speed, it changes the speed by the individual’s flight experience and group of flight dynamic adjustment [16]. Particle swarm optimization algorithm is a kind of self-adaptive random algorithm based on group hunting strategy, which is an algorithm of simple implementation and fast convergence with few parameters. At present, although the PSO algorithm has some limitations, it can be used after some appropriate improvements [15,17].

In this case, we set the search space in D dimensions with a total of *N* particles. The *i*th particle position is represented as $X_{i}=(x_{i1},x_{i2},\ldots ,x_{iD})$ and the *i*th particle’s position varying rate is represented as $V_{i}=(v_{i1},v_{i2},\ldots ,v_{iD})$. The position of each individual particle changes as follows

(10) $$v_{id}(t+1)=\omega \cdot v_{id}(t)+c_{1}\cdot r_{1}[p_{id}(t)-x_{id}(t)]+c_{2}\cdot r_{2}\cdot [p_{gd}(t)-x_{id}(t)]$$

(11) $$x_{id}(t+1)=x_{id}(t)+v_{id}(t+1)$$

where $c_{1}$, $c_{2}$ are positive constants which are called as the acceleration factors, $r_{1}$, $r_{2}$ are random numbers between [0, 1], ω is called the inertial factor, *i* is the *i*th particle (1≤i≤N), and *d* is the dimension of each particle (1≤d≤D). The initial position and speed of particle swarm are randomly generated, and are iterated according to Equations (10) and (11). The Improved Particle Swarm Optimization (IPSO) algorithm is presented based on the Basic Particle Swarm Optimization (BPSO), where the main improvements are shown as follows

1. According to the analysis of ECT imaging principle, capacitance testing equipment, and image reconstruction algorithm, a modified fitness function is presented as $F=\min(\left\|C_{M}-SG_{k}\right\|-\left\|\Delta C\right\|)$, where ‖ΔC‖ is the output trained by LS-SVM. Then, with the LS-SVM training results, one can optimize the fitness function, and compensate or eliminate the errors induced by the sensitivity matrix **S** and measuring device.
2. Based on the principle of PSO, the initial value is randomly generated, and Equation (11) is expressed as $G_{\left(t+1\right)}=G_{\left(t\right)}+v_{id}(t)$, where $v_{id}(t)$ is obtained by Equation (10). A nonlinear and dynamic adjustment method is then presented to adjust the inertia weight ω in Equation (10) as follows
  (12) $$\omega =\left\{ \begin{array}{cc} \omega_{\mathrm{int}}-(\omega_{\mathrm{int}}-\omega_{end})\cdot \frac{F-F_{\min}}{F_{avg}-F_{\min}} & F<F_{avg}\\ \omega_{\mathrm{int}} & F\geq F_{avg} \end{array}\right.$$
  where *F* is the fitness value of particle at present, $F_{avg}$ is the average fitness, and $F_{\min}$ is the minimum fitness, i.e., the fitness of the best particle. Firstly, the value of $\omega_{\mathrm{int}}$ is kept unchanged, and the value of $\omega_{end}$ is the minimum value for cumulative value of ω in each iteration (a given initial value: $\omega_{\mathrm{int}}$ = 1.2, $\omega_{end}$ = 0.8) [17]. The inertial factor is changed with the adaptation degree of each generation.
3. Population will search the extreme value that is decided by $P_{\mathrm{igbest}}$ and $P_{\mathrm{gbest}}$ after several iterations. If no better position of population than $P_{\mathrm{gbest}}$ is found in the iteration process, the algorithm will stagnate. Since the change of $P_{\mathrm{gbest}}$ can reflect the change of $P_{\mathrm{igbest}}$, the change of the best personal position of each particle can be used as the only judgment foundation for variation. In this paper, the minimal fitness value is used as the benchmark, and the fitness value at the *t*th iteration is obtain as follows
  (13) $$F_{t,avg}=\frac{1}{N}\sum_{i=1}^{N}F_{t,p_{best},i}$$
  where $F_{t,p_{best},i}$ is the best personal position of several particles at the *t*th iteration. When the condition $B:\;(F_{t+1}<F_{t,avg})$ is satisfied at the *t*+1th iteration, the optimal process is regarded as good (either too large or too small values of *K* will influence the result of genetic algorithm [18] (in this paper, *K* = 3). One can reduce the particle complexity and increase the speed of the algorithm by decreasing the particle number at this moment. When the adaptive condition does not satisfy condition *B* (i.e., $F_{t+1}=F_{t,avg}$, lasts up to three generations), the diversity of the population will be lost, and then the particle variability can be kept by increasing the number of particles.
4. One can keep the diversiform direction of movement for each particle while decreasing the computation complexity of the proposed algorithm by improving the number of particles. Firstly, we give two boundary values pop_min and pop_max. If population size has reached pop_max and it still needs to increase the particles, the population size is reduced by a particle. If population size has reached pop_min and it still needs to decrease the particles, the population size remains. The continuous generation (Consecutive Generations, CG) strategy is used to reduce the particles [18], i.e., we delete particles randomly which are not at the best locations in the current particle swarm as well as not at the optimal positions for the particle swarm. Then, the uniform mutation (Uniform Mutation, UM) [19] strategy is used to increase the particles.

$v_{np}$, $x_{np}$ and $\mathrm{pB}est_{np}$ are used as the new particle’s speed, current location, and optimal position separately in history, respectively. The updates of variation are given as follows:

(14) $$\left\{ \begin{array}{ll} \mathrm{pB}est_{np}^{d}=X_{\min}^{d}+\lambda \times (X_{\max}^{d}-X_{\min}^{d}) & d=U[1,D]\\ \mathrm{pB}est_{np}^{d}=\mathrm{pB}est_{gb}^{d} & otherwise \end{array}\right.$$

where *d* is the dimension randomly selected to mutate, and $X_{\max}^{d}$ and $X_{\min}^{d}$ are upper and lower bounds of the search space, respectively.

The flat electrode substrate is chosen according to the characteristics of the ceramic porous thermal insulation material. Therefore, based on the previous analysis of basic principle of electrical capacitance tomography and the image reconstruction algorithms (such as LBP algorithm and Landweber algorithm), an Improved Particle Swarm Optimization (IPSO) algorithm is proposed.

The proposed IPSO algorithm considers the change of sensitivity matrix **S** caused by different defect fields. The change of **S** matrix is optimized by the training of LS-SVM, which is different from the traditional ECT imaging algorithm. On this basis, the results trained by the LS-SVM are added to the fitness function of IPSO algorithm. In order to find the optimal particle, a calculating strategy of nonlinear inertial parameter ω is adopted.

At the same time the evaluation index of the particle falls into local optimum and the method of the particle to overstep the local extremum are put forward. On this basis, the IPSO algorithm for ECT image reconstruction has been proposed, which fundamentally solves the issue of iteration caused by the initial information error. Moreover, the proposed algorithm can enhance the overall searching capability and local optimum jumping capability. Furthermore, based on the IPSO algorithm, we can reconstruct the ECT image for the defect detection of adhesive layer of thermal insulation materials.

## 4. Experiment Study

A nondestructive defect detection technology for adhesive layer of thermal insulation materials is presented based on the IPSO method for ECT image reconstruction in Section 3. Three types of glue line defects (Air bubbles, Irregular defect samples, Wide glue line), which always occur in aerospace applications, are studied in this section.

**Figure 2:**
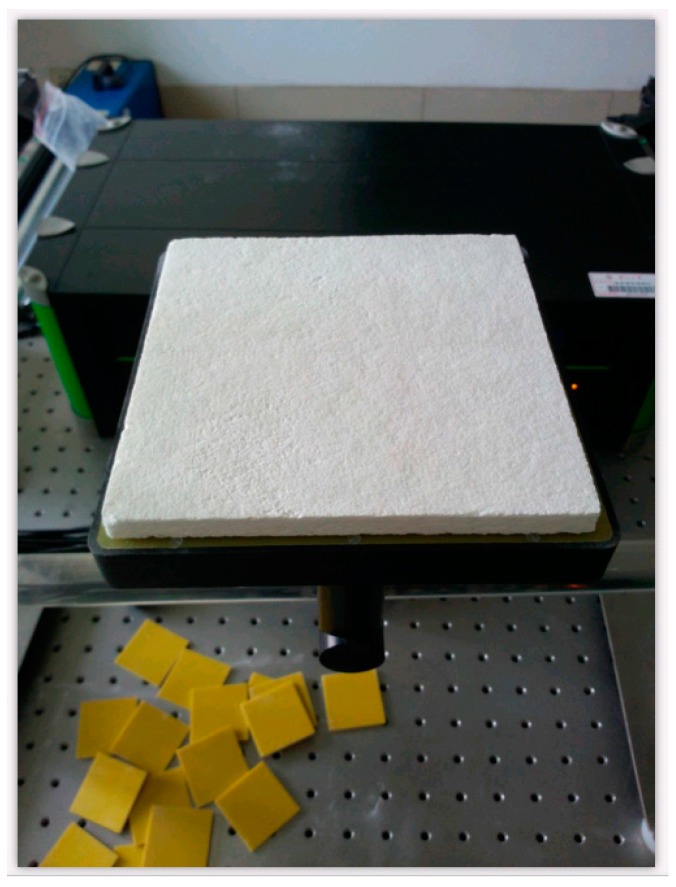
The sample without adhesion.

**Figure 3:**
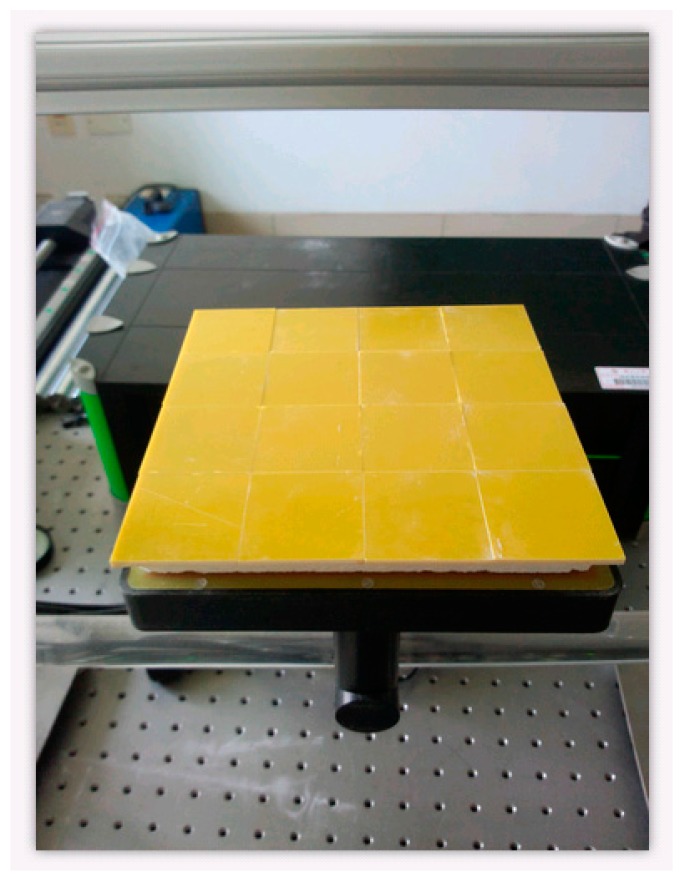
The complete sample with adhesion.

The experimental cases are as follows: 18 × 18 cm^2^ materials of porous ceramic are used for the experiments. The glue line is 16 × 16 cm^2^ epoxy resin, and the adhesive thickness is 3 mm. The full yard (full adhesive) and the empty yard (full air) are shown in Figure 2 and Figure 3.

### 4.1. Experiment Results

#### 4.1.1. Experiment for Imitation of Small Air Bubble Defects

**Figure 4:**
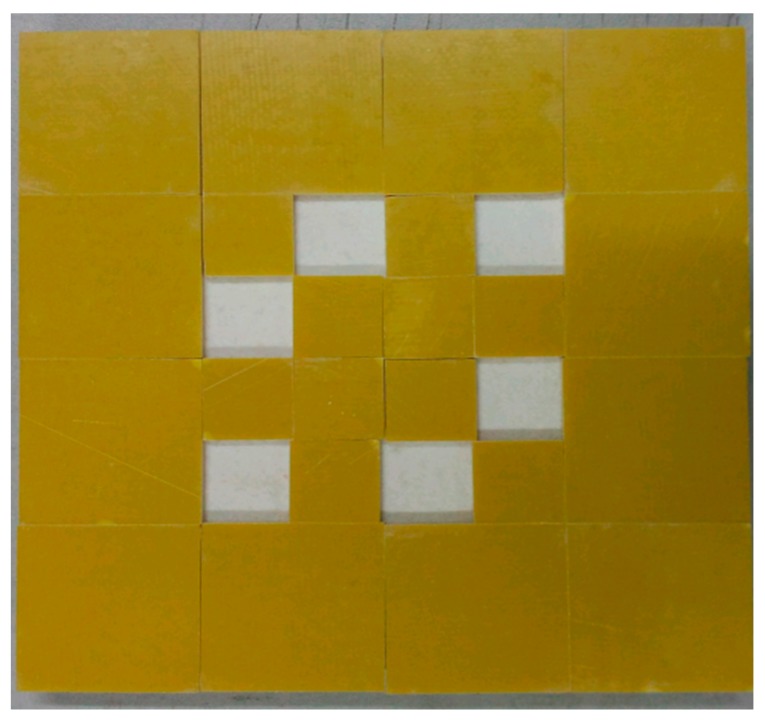
Sample 1 of defect.

We use a 16 × 16 cm^2^ epoxy resin in this experiment. To simulate the traditional defects of cementing structure such as the bubbles and lacks of plastic induced by pressing, here we consider the Sample 1 with six pieces of 2 × 2 cm^2^ square holes as the defect, as shown in Figure 4.

**Figure 5:**
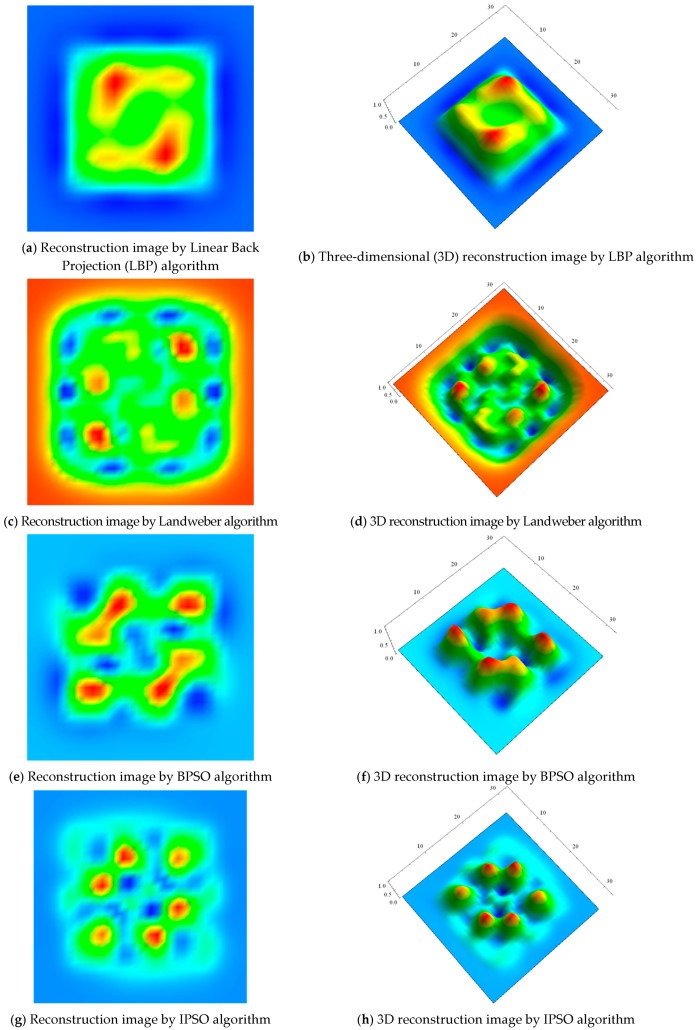
Images reconstructed by different algorithms for Sample 1.

Four algorithms of LBP, Landweber, BPSO and improved PSO algorithms are applied for defect detection of Sample 1, respectively. The simulation results are shown in Figure 5. One can find from Figure 5a,b that LBP algorithm can only roughly detect location, size, and contour information of the defect. From Figure 5c–f, the Landweber and BPSO algorithms can reflect defect, which certainly show a little better detection performance than the LBP algorithm. However, the defect marginals of reconstructed images by the three algorithms are not very clear, which makes defect edge segmentation from the reconstructed images difficult.

It is worth pointing out that, seen from Figure 5g,h, the proposed IPSO algorithm in the paper shows the significant advantages to the defect location, size, in addition, contour information of the defect can be more clearly reconstructed by the proposed IPSO algorithm. It is obvious that the defect location, size, and contour information of the defect can be more clearly reconstructed by the proposed IPSO algorithm than the other three algorithms.

#### 4.1.2. Experiment for Rubber Fracture Defects

**Figure 6:**
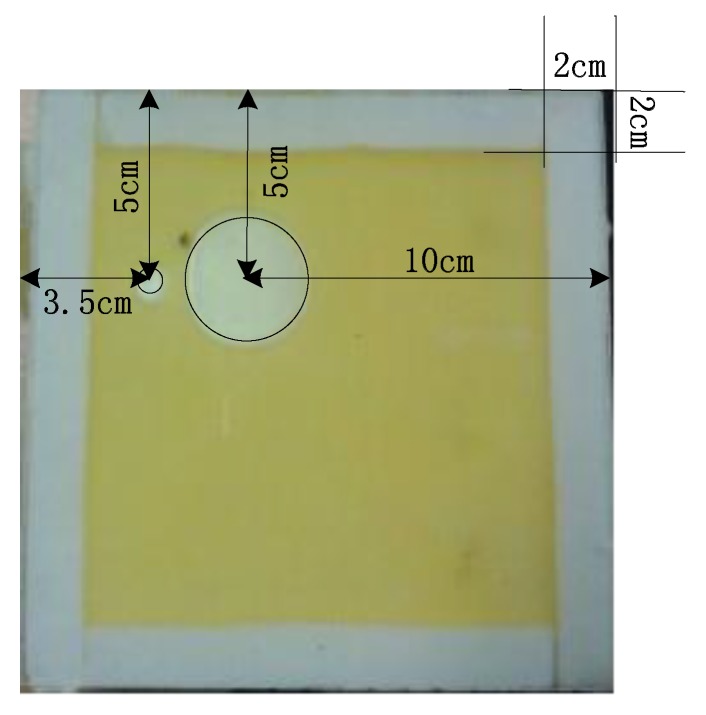
Sample 2 of defect.

Another type defect, which has two bubbles in glue line with one large and another small, as shown in Figure 6, is considered in this subsection. We call the defect as Sample 2. In order to assess the quality of image easily, two defects are separately replaced by two small circles with the areas of 7 cm^2^ and 1 cm^2^, respectively. The rubber block is surrounded by foam rubber with 2 cm wide and 16 cm length foam (the permittivity of foam rubber is similar to the permittivity of air).

**Figure 7:**
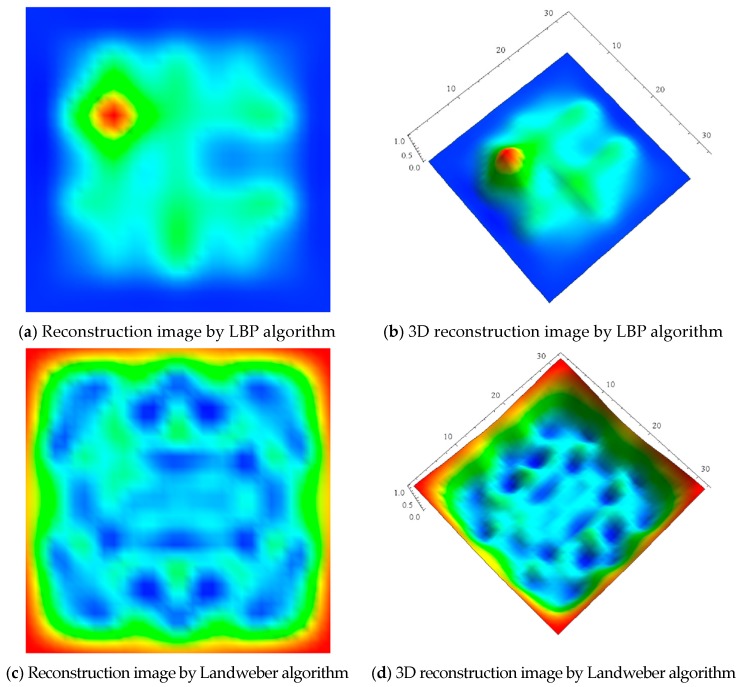

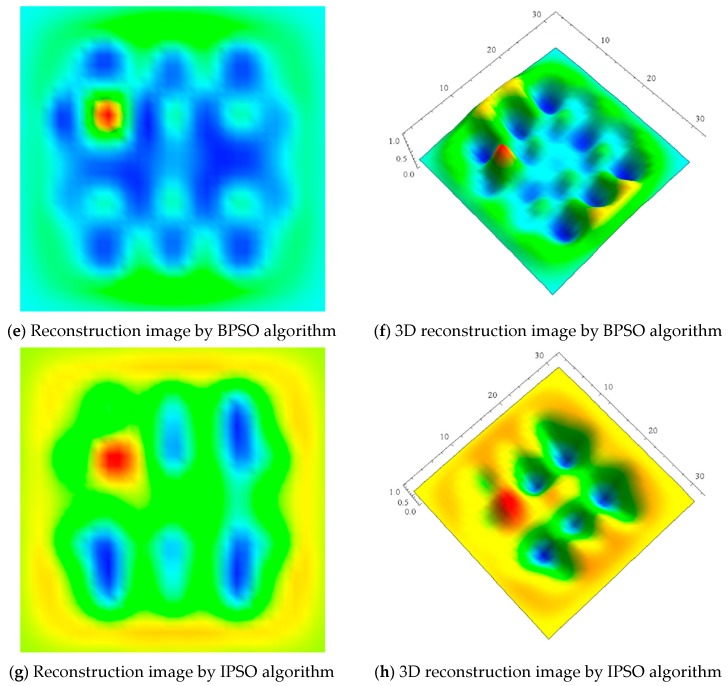
Images reconstructed by different algorithms for Sample 2.

LBP, Landweber, BPSO, and the proposed IPSO algorithms are applied for the defect detection of Sample 2. The experimental results are shown in Figure 7. It can be seen from Figure 7 that when compared with Figure 7a–f using LBP, Landweber and BPSO algorithms, the performance of reconstructed images Figure 7g,h, using the proposed IPSO are significantly improved and more detailed information of defects, such as the size, shape, edge, etc. are displayed clearly, which demonstrates the effectiveness and superiority of the proposed algorithm.

In addition, it is worth pointing out that, from all of the subfigures in Figure 7, all the 4 algorithms cannot be able to detect the smaller defect, which makes the smaller one missed in the reconstructed images. Our analysis of the results may be that the reconstructed images of the smaller defect are submerged by the ones of the large defect. Besides, to the best of our knowledge, at present, small defect below 1 cm^2^ cannot be detected by using current detection algorithms including our proposed algorithm.

#### 4.1.3. Experiment for Rubber Fracture Defects

**Figure 8:**
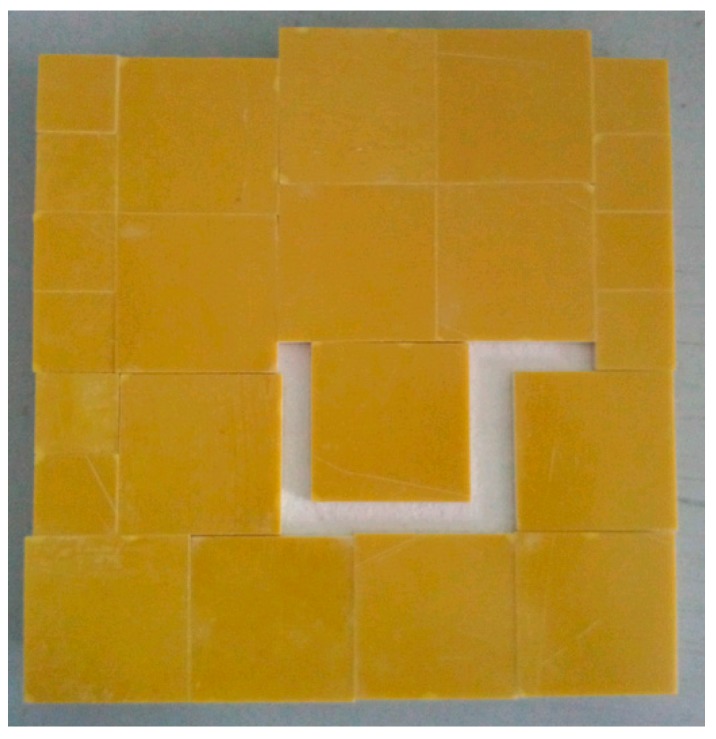
Sample 3 of defect.

To simulate the traditional defects of cemented structure, e.g., the fracture of adhesive layers, u slot defect with the entire length being 15 cm and the width 5 mm, as shown in Figure 8, is considered. We call the defect as Sample 3.

**Figure 9:**
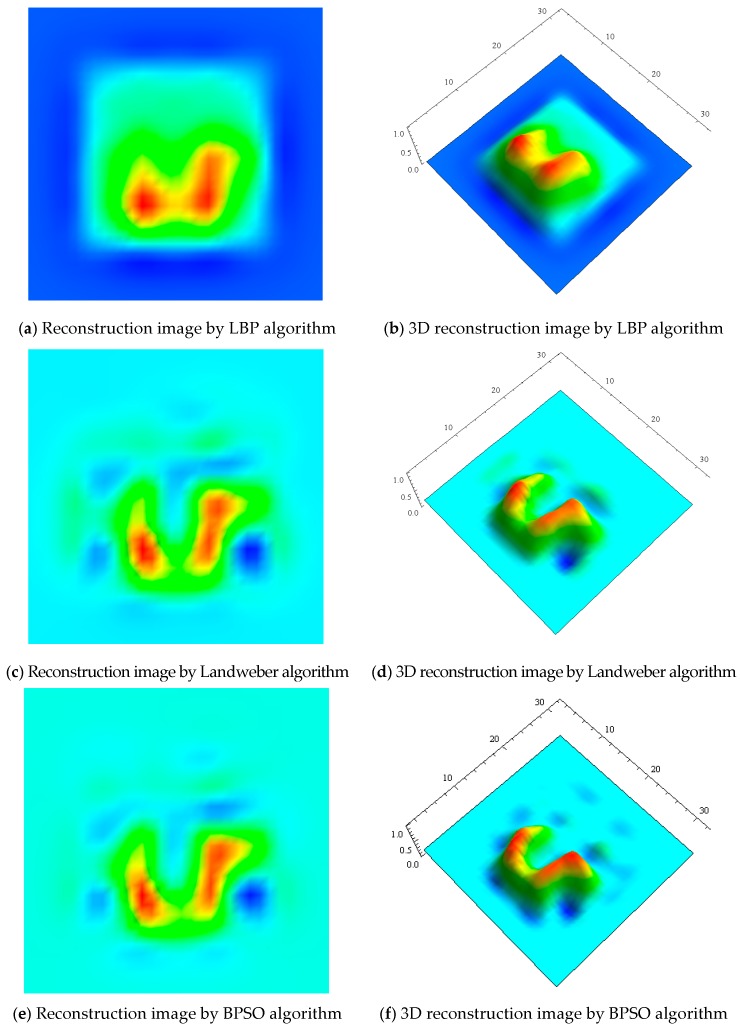

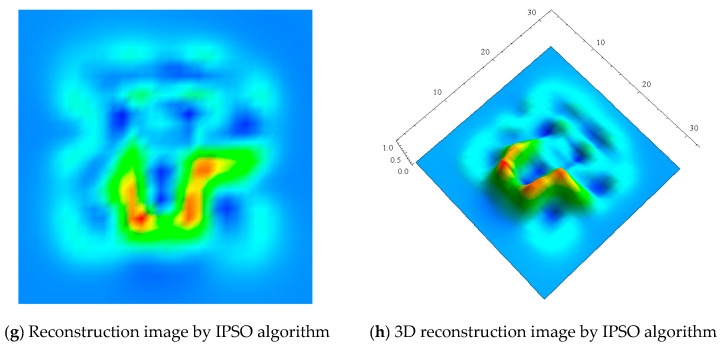
Images reconstructed by different algorithms for Sample 2.

Similarly, LBP, Landweber, BPSO and the proposed IPSO algorithms are applied to detect the defects of Sample 3. The experimental results are shown in Figure 9. It can be seen from Figure 9 that, when compared with Figure 9a–f using LBP, Landweber, and BPSO algorithms, the effect of reconstructed images Figure 9g,h using the proposed IPSO are significantly improved, and more detailed information of defects, such as the size, shape, edge, etc. are displayed more clearly, which also demonstrates the effectiveness and the superiority of the proposed IPSO algorithm.

It is worth noting that because the capacitance distribution from 12-electrode capacitance sensors is used, the quality of reconstructed images is not effective to some defects with sizes being below the cm-level. The accuracy of reconstructed images is influenced by the less original data of capacitance to a certain extent. However, in the real applications, the destructive effect of below the cm-level’s defect of adhesive layer of thermal insulation materials is far less than that of the above cm-level’s defect. Obviously, for the defect detection of defect area greater than 1 cm^2^, the IPSO algorithm can achieve significantly better performance than the classical algorithms.

### 4.2. Evaluation of Algorithms by Experiment

In this subsection, we will further evaluate the performance of the four algorithms. Two evaluation criteria will be used in this paper: the image correlation $I_{c}$, which is the similarity degree between the reconstruction image and the test object image, and the relative image error $I_{e}$. They are shown as follows

(15) $$I_{c}=\frac{\sum_{i=1}^{m}\left[(e_{i}^{\left(k\right)}-{\bar{E}}^{\left(k\right)})(e_{i}^{0}-{\bar{E}}^{0})\right]}{\sum_{i=1}^{m}\sqrt{{\left(e_{i}^{\left(k\right)}-{\bar{E}}^{\left(k\right)}\right)}^{2}}\sum_{i=1}^{m}\sqrt{{\left(e_{i}^{0}-{\bar{E}}^{0}\right)}^{2}}}$$

(16) $$I_{e}=\frac{\sqrt{\sum_{i=1}^{m}{\left(e_{i}^{\left(k\right)}-e_{i}^{0}\right)}^{2}}}{\sqrt{{\left({\bar{E}}^{0}\right)}^{2}}}$$

where $e_{i}^{\left(k\right)}$ is the *i*th element in the final reconstructed image $E^{\left(k\right)}$, ${\bar{E}}^{\left(k\right)}$ is the average of pixels in the $E^{\left(k\right)}$ space, and $e_{i}^{0}$ is the *i*th element in the original simulated image $E^{0}$. The clearer the reconstruction image is, if the lower $I_{e}$ is and the higher $I_{c}$ is [19].

The computing results under the four algorithms are shown in Table 1 and Table 2. Table 1 shows the image correlation coefficients of the reconstructed images for Sample 1, Sample 2, and Sample 3 using LBP, Landweber, BPSO and IPSO, respectively. Table 2 shows the errors of the reconstructed images of Sample 1, Sample 2 and Sample 3 based on LBP, Landweber, BPSO and IPSO, respectively. Obviously, the simulation results in Table 1 and Table 2 demonstrate that the proposed IPSO scheme has higher significant effect for defect image reconstruction than the other three algorithms.

## 5. Conclusions and Future Work

This paper develops a novel non-destructive method for defect detection of adhesive layer of thermal insulation materials based on the proposed IPSO algorithm. In the method, the least squares support vector machine is utilized at first for data processing of measured capacitance values, and then the improved PSO algorithm is proposed for the optimization of image reconstruction. Simulation and experiment results demonstrate that when compared with the traditional algorithms such as LBP algorithm and Landweber algorithm, the proposed IPSO algorithm can display the defect information including size, shape, and edge of the defects more clearly. Future work will be implemented to further identify the feature of defects.

## Figures and Tables

**Table 1:** Image correlation $I_{c}$ (%) by different algorithms.

	Sample 1	Sample 2	Sample 3
LBP	0.5680	0.6087	0.4787
Landweber	0.4680	0.6829	0.6853
BPSO	0.7648	0.6946	0.6992
IPSO	0.8484	0.7737	0.7712

**Table 2:** Relative image error $I_{e}$ by different algorithms.

	Sample 1	Sample 2	Sample 3
LBP	2.1927	1.2625	3.6358
Landweber	2.3209	0.8923	4.7879
BPSO	2.1230	0.9321	4.5827
IPSO	1.0999	0.8317	3.5059
